# An *Acinetobacter* non-*baumannii* Population Study: Antimicrobial Resistance Genes (ARGs)

**DOI:** 10.3390/antibiotics10010016

**Published:** 2020-12-26

**Authors:** Adam Baraka, German M. Traglia, Sabrina Montaña, Marcelo E. Tolmasky, Maria Soledad Ramirez

**Affiliations:** 1 Center for Applied Biotechnology Studies, Department of Biological Science, College of Natural Sciences and Mathematics, California State University Fullerton, Fullerton, CA 92831, USA; abaraka1@hotmail.com (A.B.); mtolmasky@fullerton.edu (M.E.T.); 2 Departamento de Desarrollo Biotecnológico, Instituto de Higiene, Facultad de Medicina, Universidad de la República, MVD, Montevideo 11800, Uruguay; gertra13a@gmail.com; 3 Laboratorio de Bacteriología Clínica, Departamento de Bioquímica Clínica, Hospital de Clínicas José de San Martín, Facultad de Farmacia y Bioquímica, Buenos Aires 1120, Argentina; sabri.mon@hotmail.com

**Keywords:** *Acinetobacter* non-*baumannii*, antimicrobial resistance, resistome

## Abstract

*Acinetobacter* non-*baumannii* species are becoming common etiologic agents of nosocomial infections. Furthermore, clinical isolates belonging to this group of bacteria are usually resistant to one or more antibiotics. The current information about antibiotic resistance genes in the different *A.* non-*baumannii* species has not yet been studied as a whole. Therefore, we did a comparative study of the resistomes of *A.* non-*baumannii* pathogens based on information available in published articles and genome sequences. We searched the available literature and sequences deposited in GenBank to identify the resistance gene content of *A. calcoaceticus*, *A. lwoffii*, *A. junii*, *A. soli*, *A. ursingii*, *A. bereziniae*, *A. nosocomialis*, *A. portensis*, *A. guerrae*, *A. baylyi*, *A. calcoaceticus*, *A. disperses*, *A. johnsonii*, *A. junii*, *A. lwoffii*, *A. nosocomialis*, *A. oleivorans*, *A. oryzae*, *A. pittii*, *A. radioresistens*, and *A. venetianus.* The most common genes were those coding for different β-lactamases, including the carbapenemase genes *bla*_NDM-1_ and *bla*_OXA-58_. *A. pittii* was the species with the most β-lactamase resistance genes reported. Other genes that were commonly found include those encoding some aminoglycoside modifying enzymes, the most common being *aph(6)-Id*, *ant(3″)-IIa*, and *aph(3″)-Ib*, and efflux pumps. All or part of the genes coding for the AdeABC, AdeFGH, and AdeIJK efflux pumps were the most commonly found. This article incorporates all the current information about *A.* non-*baumannii* resistance genes. The comparison of the different resistomes shows that there are similarities in the genes present, but there are also significant differences that could impact the efficiency of treatments depending on the etiologic agent. This article is a comprehensive resource about *A.* non-*baumannii* resistomes.

## 1. Introduction

The genus *Acinetobacter* is a very diverse group of bacteria considered one of the most troublesome opportunistic nosocomial pathogens. It comprises 62 species with assigned names (http://www.bacterio.net/a/acinetobacter.html). *A. baumannii* is responsible for most nosocomial infections and has an intrinsic ability to acquire resistance to all available antibiotics. The problems caused by this bacterium led World Health Organization (WHO) experts and researchers from the Division of Infectious Diseases at the University of Tübingen (Germany) to include it in the critical priority group. Furthermore, the rise of carbapenem-resistant *Acinetobacter* led to its placement within a group of five pathogens considered as urgent threats to human health by the Center for Disease Control [1]. Although in the past decades the vast majority of *Acinetobacter* infections used to be caused by the species *baumannii*, in the past few years numerous other *Acinetobacter* species became commonly identified as causative agents of nosocomial infections. *A. calcoaceticus*, *A. lwoffii*, *A. junii*, *A. soli*, *A. ursingii*, *A. bereziniae* and *A. nosocomialis* are also becoming common culprits of hospital infections [2,3]. Despite their diversity, a common characteristic of these species is the presence of multiple antibiotic resistance genes. Likewise, multiple antibiotic resistance mechanisms have been documented in other *Acinetobacter* species (*A. lwoffii*, *A. johnsonii*, *A. junnii*, *A. nosocomialis* and *A. pittii*) mainly recovered from soil and wastewater, as well as vegetables and meats. These species are important environmental reservoirs of resistance genetic determinants, which could later give rise to clinically relevant strains [2,3,4,5,6,7].

In this work, we conduct a comprehensive analysis of reported antibiotic resistance genes in *A.* non-*baumannii* species. Other genes found in a lower number of isolates were those coding for resistance to trimethoprim (*dfrA*-like), macrolide (*ereA2*, *ermB*), colistin (*mcr-1*, *mcr-4*), fluoroquinolone (*qnrD1*, *qnrS1*), rifampicin (*arr-2*), chloramphenicol (*cat*-like), lincosamide (*InuF*, *InuG*) and tetracycline (*tet*-like).

## 2. Results and Discussion

**Figure 1:**
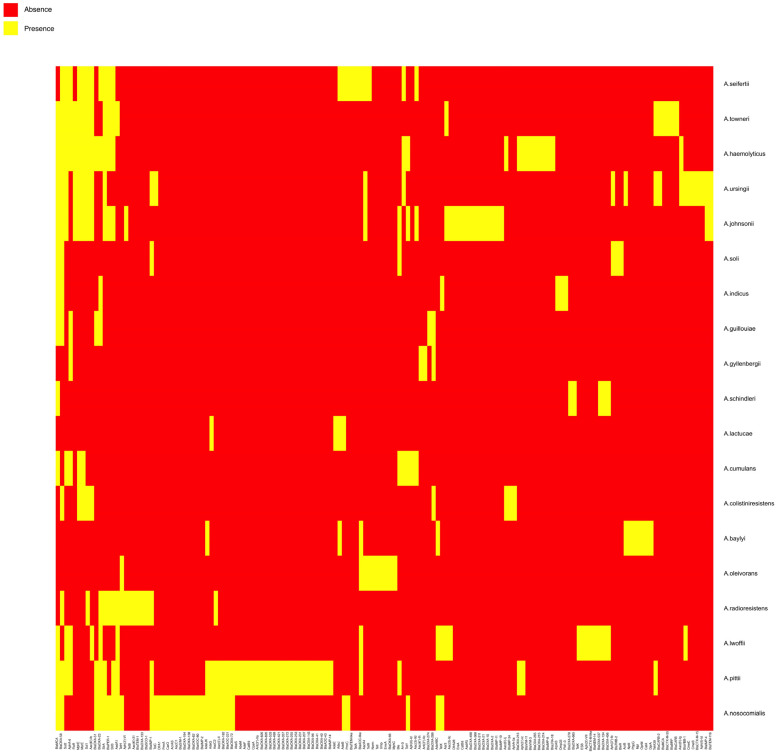
*A*. non-*baumannii* species which carry three or more ARGs as found in the literature. Heatmap showing species that harbor of three or more ARGs. Yellow, presence of the gene; red, absence of the gene in the corresponding species. A complete listing including *A*. non-*baumannii* species that carry any number of ARGs can be found in Table S1 and Figure S1.

A summary of all *A.* non-*baumannii* species harboring potential antibiotic resistance genes (ARGs) reported in the literature is shown in Table 1. A detailed listing of the individual ARGs identified in each strain is shown in Table S1. The vast majority of ARGs code for β-lactamases of different classes (*n* = 153 for 31 isolates). The highest represented β-lactamase genes were *bla*_NDM-1_ and *bla*_OXA-58_ (14 and 12 strains, respectively) (Table S1). NDM-1 is a troublesome metallo-β-lactamase originally identified in a carbapenem-resistant *Klebsiella pneumoniae* isolate that, except for monobactams, confers resistance to all other β-lactam antibiotics [8,9,10]. The *bla*_OXA-58_ gene was first found in a multiresistant *A. baumannii* isolated in a hospital in France [11]. OXA-58 belongs to a group of enzymes with a reduced number of variants. *A. baumannii* strains carrying this enzyme showed different levels of resistance to carbapenems [10]. *A. pittii* was the species with the most β-lactamase resistance genes reported (*n* = 28), followed by *A. nosocomialis* (*n* = 15) and *A. haemolyticus* (*n* = 14) (Figure 1, Figure S1, and Table S1). 

The second most abundant resistance mechanism was that mediated by aminoglycoside modifying enzymes (*n* = 77 for 20 species, Table 1), *aphA6* and *strA* being the most reported genes. Aminoglycosides are bactericidal antibiotics used to treat a wide range of bacterial infections, including those caused by *Acinetobacter* [12,13]. The most common mechanism of resistance to aminoglycosides is the enzymatic inactivation of the antibiotic molecule. Aminoglycoside modifying enzymes catalyze the transfer of acetyl, phosphate, or nucleotidyl groups to the –OH or –NH2 groups of the 2-deoxystreptamine or the sugar moieties of the molecule [14]. 

The third most abundant mechanism was the efflux pumps, i.e., cellular systems that can export compounds usually without specificity. This mechanism specifies resistance through detoxification [15,16]. A total of 38 antibiotic efflux pump genes were identified in 12 isolates (Figure 1, Figure S1, and Table S1). The resistance-nodulation-cell division (RND) family efflux systems AdeABC and AdeIJK were the most common efflux pumps present in *A.* non-*baumannii* species. Both efflux systems can expel a broad range of antibiotics [17,18,19]. In the case of *A. baumannii*, all isolates studied to date carry AdeIJK, and a majority, but not all, carry AdeABC [19]. *A. pittii* is the species with the highest variety ARGs (*n* = 46), followed by *A. nosocomialis* (*n* = 35), *A. johnsonii* (*n* = 33), *A.* and *A. haemolyticus* (*n* = 28). Thirty-four *A.* non-*baumannii* species did not carry any of the tested ARGs (Table S1).

Since the nucleotide sequence data banks include an excess of information when compared to that already published in scientific articles, another set of analyses was carried out using the Basic Local Alignment Search Tool (BLAST) software and the data from the databases CARD-RGI, ARG-ANNOT, and ResFinder. There were 1457 complete *A.* non-*baumannii* genomes in GenBank as of June 15, 2020. Each one of them was compared with the databases CARD-RGI, ARG-ANNOT and ResFinder using BLAST. Comparisons to information in the ARG-ANNOT database produced 9676 hits corresponding to efflux pumps genes in 1404 genomes. Genes related to AdeABC, AdeFGH and AdeIJK were the efflux pumps most commonly present, and AdeFGH and AdeIJK were those found more often in a complete version (Figure S2 and Table S2). The second group of predominant genes was β-lactamase resistance genes (*n* = 1574 for 963 genomes) (Figure S2 and Table S2). A total of 245 *A. pittii*, 187 *A.* sp., and 143 *A. nosocomialis* isolates harbored β-lactamase resistance genes. The *bla*_OXA-421_ and *bla*_ADC-4_ genes were the highest represented β-lactamases. The species with the highest ARG content were *A. pitti* (*n* = 3841), *A.* sp. (*n* = 2915), and *A. nosocomialis* (*n* = 2277). 

Aminoglycoside modifying enzyme coding genes were the third most abundant group of resistance determinants; 1084 genes being found in 599 genomes in the database. The species with the most aminoglycoside resistance genes were *A. pitti*, *A.* sp, *A. indicus* and *A. haemolyticus.* The most identified genes were *aph(6)-Id*, *ant(3″)-IIa* and *aph(3″)-Ib* (Figure S2). 

No ARGs were identified in *A. nectaris*, *A. marinus*, *A. equi*, *A. larvae*, *A. qingfengensis* and *A. boissieri* by BLAST comparisons or the literature search. In the cases of *A. albensis*, *A. bouvetii*, *A. brisouii*, *A. celticus*, *A. harbinensis*, *A. junii*, *A. kookii*, *A. pragensis* and *A. pseudolwoffii*, no ARGs were identified in the literature. Still, at least one resistance-nodulation-cell division (RND) antibiotic efflux pump gene was identified by the BLAST comparisons. All adeIJK efflux pump genes were identified in *A. defluvii*, *A. junii*, *A. venetianus*, *A.halotolerans*, *A. tjernbergiae*, and *A. wuhouensis*. All adeFGH efflux pump genes were found in *A. courvalinii*. Besides the efflux pump, other ARGs genes were detected in *A. bouvetii*, *A. courvalinii*, *A. defluvii*, *A. junni*, *A. pseudolwoffii*, *A. venetianus* and *A. wuhouensis*).

The BLAST comparison permitted us to detect genes and the number of allelic variants (n) that are not yet reported in *A.* non-*baumannii* publications (Figures S1 and S2, Tables S1 and S2). The β-lactamase coding genes found are *bla*_ADC-like_ (*n* = 22), *bla*_OXA-like_ (*n* = 46), *bla*_KPC-1_, *bla*_CTX-M-65_, *bla*_NDM-3,_ *bla*_PER-2,_ *bla*_CARB-like_ (*n* = 4), *bla*_TEM-4,_ *bla*_VIM-4,_ *bla*_VEB-7,_ *bla*_GES-7_. The aminoglycoside modifying enzyme coding genes identified were *AAC(6′)-*like (*n* = 12), *AAC(3)-*like (*n* = 3), *aadA*-like (*n* = 5), *ANT(2″)-Ia*, *ANT(3″)-IIb*, *APH(4)-Ia*, *APH(3′)-*like (*n* = 3). In addition, we identified genes that code for resistance to trimethoprim (*dfrA*-like (*n* = 7)), macrolide (*ereA2*, *ermB*), colistin (*mcr-1*, *mcr-4*), fluoroquinolone (*qnrD1*, *qnrS1*), rifampicin (*arr-2*), chloramphenicol (*cat*-like (*n* = 7)), lincosamide (*InuF*, *InuG*) and tetracycline (*tet*-like (*n* = 4)). Among the efflux pump related genes, we identified *adeH*, *adeL*, *adeN*, *adeR*, *and adeS.*

We also found ARGs described in *A.* non-*baumannii* publications that were not identified by the BLAST comparisons (Tables S1 and S2). The β-lactamase coding genes found are *bla*_ADC-221_, *bla*_IMP-like_ (*n* = 4), *bla*_VIM-like_ (*n* = 2), *bla*_CTX-M-15,_ *bla*_NDM-14,_ *bla*_CARB-2_, *bla*_SCO-1,_ *bla*_OXA-like_ (*n* = 29), *bla*_TEM-like_ (*n* = 3). The aminoglycoside modifying enzyme coding genes detected were *aphA*-like (*n* = 4), *nptII*, *strA*, *cpaA*, *aacC2*, *aacC.* Other genes identified were those coding for resistance to trimethoprim (*dfrA*-like (*n* = 2)), chloramphenicol (*cat*-like (*n* = 3)), macrolide (*ereA*, *mphC*, *mphE*, *pld1-3*), fluoroquinolone (*acrB*), tetracycline (*tet*-like (*n* = 4)). Among the efflux pump related genes, we identified *AcrAB*, *adeE*, *dprA*, *cmeB*, *cmeC*, *craA*, *tolC*, *norm*, *emrA*, *pmrA*, *ump* and *mpl.*

## 3. Materials and Methods

### 3.1. Literature Search

All known *Acinetobacter* species, as listed in the List of Prokaryotic names with Standing in Nomenclature [20], were used in a PubMed literature search. Two species, (*A. portensis* and *A. guerrae*), recently discovered by Caravalheira et. al. [21] were also included. The search was conducted by inputting the full name of each species with the All Fields selection in the PubMed database and then identifying the antibiotic resistance genes described in each article. CARD-RGI software [22] was used to verify the antibiotic resistance nature of genes that were not well defined in the publications. 

### 3.2. Genome Sequences Collection and Antibiotic Resistance Genes Prediction

All complete *A.* non-*baumannii* genome nucleotide sequences were downloaded from GenBank NCBI. Predictive identification of antibiotic resistance genes (ARGs) was performed using the BLASTp, the CARD-RGI [22], ARG-ANNOT [23] and ResFinder [24] software databases. For ARGs prediction, 70% coverage, 95% amino acid identity and <10^−6^ e-value were used as the BLASTp parameters. Data prediction using the three databases were integrated for clustering analysis. Clustering analysis was carried out using the *hclust* and *dist* R package [25]. The Euclidean method was used as distance method. The agglomerative method was used as Hierarchical clustering method. Within the agglomerative methods, the complete-linkage algorithm was used for hierarchical clustering analysis. Agglomerative methods were reported as the most adequate to analyze the presence and absence of genes over genomes [26].

## 4. Conclusions 

The importance of *A.* non-*baumannii* as pathogens, and the volume of information about their resistance to antimicrobials, is rapidly increasing. This article describes an initial study of the resistome of *A.* non-*baumannii* pathogens. The impact of this group of bacteria as causative agents of nosocomial infections is growing. As a consequence, while *Acinetobacter* infections are still mainly caused by *A. baumannii* [1,27], it is quite probable that other species will become as important in the future. It is already evident that multiple *A.* non-*baumannii* clinical isolates are resistant to one or more antibiotics [2,3]. The present and future efforts to design effective therapies against these pathogens require understanding their resistance profiles and genes. The comparison of existing data (literature and GenBank) about resistance genetic determinants showed similarities and significant differences that could impact the efficiency of treatments depending on the species that originate each infection.

## Figures and Tables

**Table 1:** Number of antibiotic resistance genes (ARGs) in *Acinetobacter* non-*baumannii* isolates reported in the literature.

Number of ARG in *Acinetobacter* non-*baumannii* Isolates Reported in the Literature																
β-lactamases	Aminoglycosides	Efflux Pump Genes	Sulfonamides	Tetracyclines	Macrolides	ABC-F	Rifampicin	Florfenicol	Bleomycin	Chloramphenicol	Carbapenem (CarO Specific)	OMP	Fluoroquinolone	PBPs	Pmr	Trimhoprim
*A. baylys* (*n* = 1)	*A. baylys* (*n* = 1)	*A. apis* (*n* = 1)	*A. colistiniresistens* (*n* = 1)	*A. indicus* (*n* = 1)	*A. colistiniresistens* (*n* = 1)	*A. colistiniresistens* (*n* = 1)	*A. cumulans* (*n* = 1)	*A. haemolyticus* (*n* = 1)	*A. cululans* (*n* = 1)	*A. baylyi* (*n* = 1)	*A. apis* (*n* = 1)	*A. apis* (*n* = 1)	*A. baylyi* (*n* = 1)	*A. baylyi* (*n* = 1)	*A. nosocomialis* (*n* = 1)	*A. nosocomialis* (*n* = 1)
*A. bereziniae* (*n* = 1)	*A. calcoaceticus* (*n* = 1)	*A. baylys* (*n* = 6)	*A. cumulans* (*n* = 1)	*A. lwoffii* (*n* = 1)	*A. cumulans* (*n* = 1)	*A. cumulans* (*n* = 1)	*A. haemolyticus* (*n* = 1)	*A. johnsonii* (*n* = 1)	*A. johnsonii* (*n* = 1)	*A. johnsonii* (*n* = 1)	*A. nosocomialis* (*n* = 1)	*A. indicus* (*n* = 1)	*A. ursingii* (*n* = 1)	*A. indicus* (*n* = 1)	*A. seifertii* (*n* = 1)	*A. johnsonii* (*n* = 1)
*A. beijerinckii* (*n* = 1)	*A. colistiniresistens* (*n* = 3)	*A. cumulans* (*n* = 1)	*A. gandensis* (*n* = 1)	*A. nosocomialis* (*n* = 2)	*A. haemolyticus* (*n* = 1)	*A. haemolyticus* (*n* = 1)	*A. johnsonii* (*n* = 1)	*A. seifertii* (*n* = 1)	*A. pittii* (*n* = 1)	*A. modestus* (*n* = 1)						
*A. bohemicus* (*n* = 1)	*A. cumulans* (*n* = 3)	*A. haemolyticus* (*n* = 1)	*A. haemolyticus* (*n* = 2)	*A. oleivorans* (*n* = 3)	*A. indicus* (*n* = 1)	*A. johnsonii* (*n* = 1)	*A. lwoffii* (*n* = 1)	*A. towneri* (*n* = 1)	*A. soli* (*n* = 2)	*A. pittii* (*n* = 1)						
*A. calcoaceticus* (*n* = 3)	*A. gerneri* (*n* = 2)	*A. johnsonii* (*n* = 1)	*A. johnsonii* (*n* = 2)	*A. pitii* (*n* = 1)	*A. johnsonii* (*n* = 2)	*A. seifertii* (*n* = 1)	*A. seifertii* (*n* = 1)	*A. ursingii* (*n* = 1)								
*A. chinensis* (*n* = 1)	*A. guillouiae* (*n* = 1)	*A. lactucae* (*n* = 4)	*A. lwoffii* (*n* = 1)	*A. radioresistens* (*n* = 2)	*A. oleivorans* (*n* = 1)	*A. towneri* (*n* = 1)	*A. towneri* (*n* = 1)									
*A. colistiniresistens* (*n* = 3)	*A. gyllenbergii* (*n* = 3)	*A. lwoffii* (*n* = 3)	*A. pittii* (*n* = 1)	*A. seifertii* (*n* = 2)	*A. seifertii* (*n* = 1)	*A. ursingii* (*n* = 1)	*A. ursingii* (*n* = 1)									
*A. cumulans* (*n* = 1)	*A. haemolyticus* (*n* = 7)	*A. nosocomialis* (*n* = 8)	*A. radioresistens* (*n* = 1)	*A. towneri* (*n* = 1)	*A. towneri* (*n* = 1)											
*A. dijkshoorniae* (*n* = 3)	*A. johnsonii* (*n* = 9)	*A. oleivorans* (*n* = 3)	*A. seifertii* (*n* = 2)	*A. ursingii* (*n* = 1)	*A. ursingii* (*n* = 1)											
*A. disperusus* (*n* = 1	*A. lwoffii* (*n* = 5)	*A. pittii* (*n* = 5)	*A. towneri* (*n* = 2)													
*A. gandensis* (*n* = 2)	*A. nosocomialis* (*n* = 7)	*A. seifertii* (*n* = 2)	*A. ursingii* (*n* = 2)													
*A. guillouiae* (*n* = 6)	*A. oleivorans* (*n* = 1)	*A. ursingii* (*n* = 3)														
*A. gyllenbergii* (*n* = 1)	*A. parvus* (*n* = 1)															
*A. haemolyticus* (*n* = 14)	*A. pittii* (*n* = 9)															
*A. indicus* (*n* = 3)	*A. radioresistens* (*n* = 6)															
*A. johnsonii* (*n* = 13)	*A. rudis* (*n* = 1)															
*A. kyonggiensis* (*n* = 1)	*A. seifertii* (*n* = 6)															
*A. lwoffii* (*n* = 9)	*A. soli* (*n* = 1)															
*A. nosocomialis* (*n* = 15)	*A. ursingii* (*n* = 5)															
*A. oleivorans* (*n* = 2)	*A. towneri* (*n* = 5)															
*A. parvus* (*n* = 1)																
*A. pitti* (*n* = 28)																
*A. proteolyticus* (*n* = 1)																
*A. radioresistens* (*n* = 7)																
*A. schindleri* (*n* = 6)																
*A. seifertii* (*n* = 5)																
*A. soli* (*n* = 4)																
*A. tandoii* (*n* = 1)																
*A. towneri* (*n* = 8)																
*A. ursingii* (*n* = 8)																
*A. variabilis* (*n* = 2)																
**Total Number of ARGs**																
N = 153	N = 77	N = 38	N = 16	N = 14	N = 10	N = 7	N = 7	N = 5	N = 4	N = 4	N = 2	N = 2	N = 2	N = 2	N = 2	N = 2

The antibiotic resistance genes were identified searching PubMed. The search was carried out inputting the full name of each species with the All Fields selection, followed by identification of the antibiotic resistance genes in each article. ABC-F ATP-binding cassette ribosomal protection protein gene resistance; OMP: Outer membrane porin drug resistance genes; PBPs: penicllin-binding proteins; Pmr: phosphoethanolamine transferase.

## Data Availability

Data sharing not applicable.

## Supplementary Materials

The following are available online at https://www.mdpi.com/2079-6382/10/1/16/s1, Figure S1. Heatmap showing absence and presence of ARGs in *A.* non-*baumanii* reported in the literature. Red color indicates the absence of genes and blue color indicates the presence of gene. The clustering analysis was done using *hclust* R package. The heatmap was done using to ggplot2 R package. Figure S2. Heatmap showing absence and presence of ARGs in *A.* non-*baumannii* genomes available in the GenBank database. Red color indicates the absence of a gene and blue color indicates the presence of a gene. The GenBank Assembly Accession of the genomes used is indicating in Table S3. The clustering analysis was done using *the* *hclust* R package. The heatmap was done using the ggplot2 R package. Table S1. List of ARGs in *Acinetobacter*, excluding *A. baumannii*, reported in the literature. The presence of ARGs is indicated with color cell and the absence of ARGs is indicated with white cell. Table S2. List of ARGs in *Acinetobacter*, excluding *A. baumannii*, predicted in available genomes in GenBank Database. The presence of ARGs is indicated with 1 and the absence of ARGs is indicated with 0. Table S3. List of Code used in Figure S2.
