# Supplementary figures and images for: An Acinetobacter non-baumannii Population Study: Antimicrobial Resistance Genes (ARGs)
Source: Antibiotics (Basel). 2020 Dec 26;10(1):16. doi: 10.3390/antibiotics10010016 (PMC7823295; doi:10.3390/antibiotics10010016)

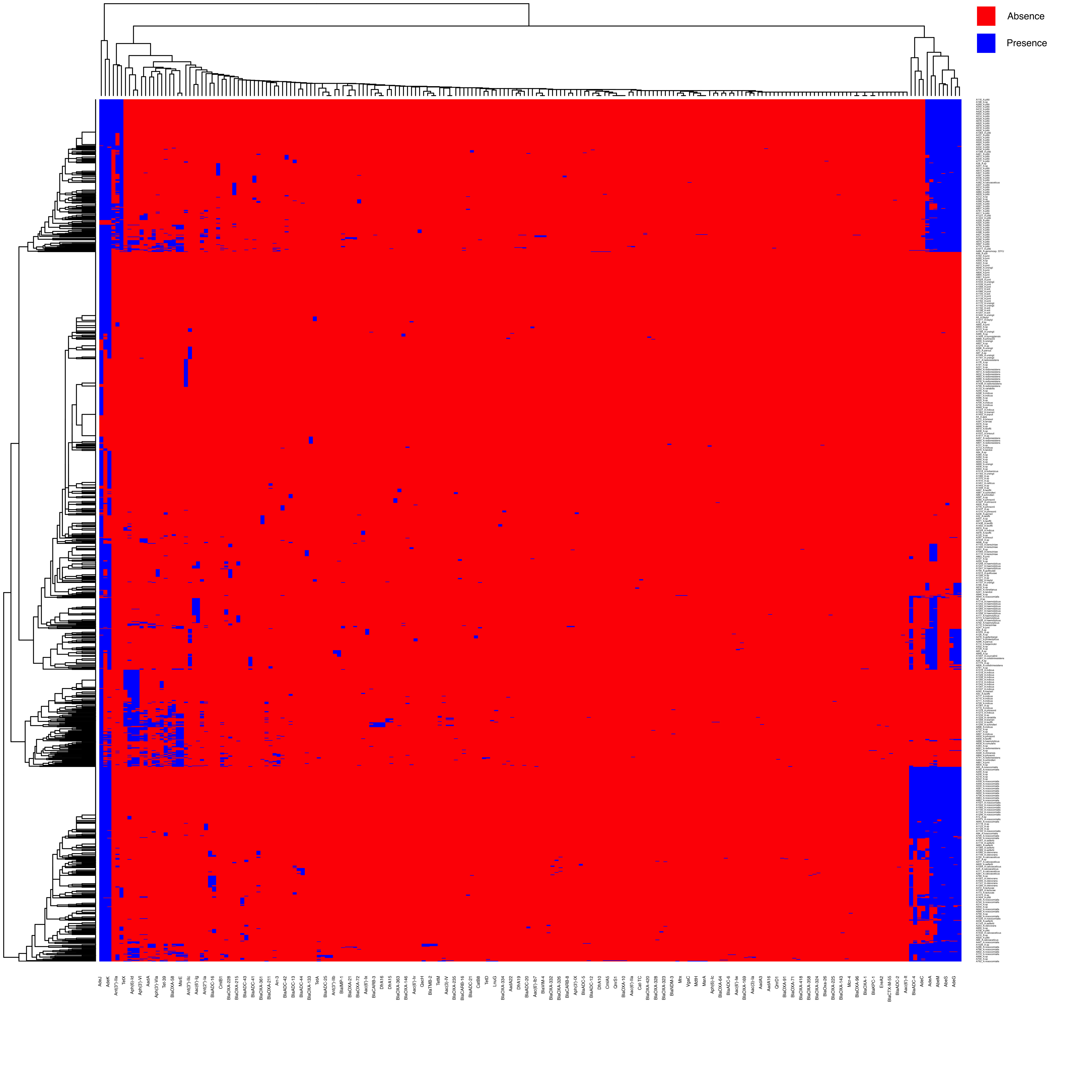

Supplement: Supplementary file 1 [file antibiotics-10-00016-s001.zip › Figure S2_genomes.pdf]
