# Supplementary material for: An Acinetobacter non-baumannii Population Study: Antimicrobial Resistance Genes (ARGs)
Source: Antibiotics (Basel). 2020 Dec 26;10(1):16. doi: 10.3390/antibiotics10010016 (PMC7823295; doi:10.3390/antibiotics10010016)
Supplement: Supplementary file 1 [file Figure S1 _Lit.pdf › Figure S1 _Lit.pdf]

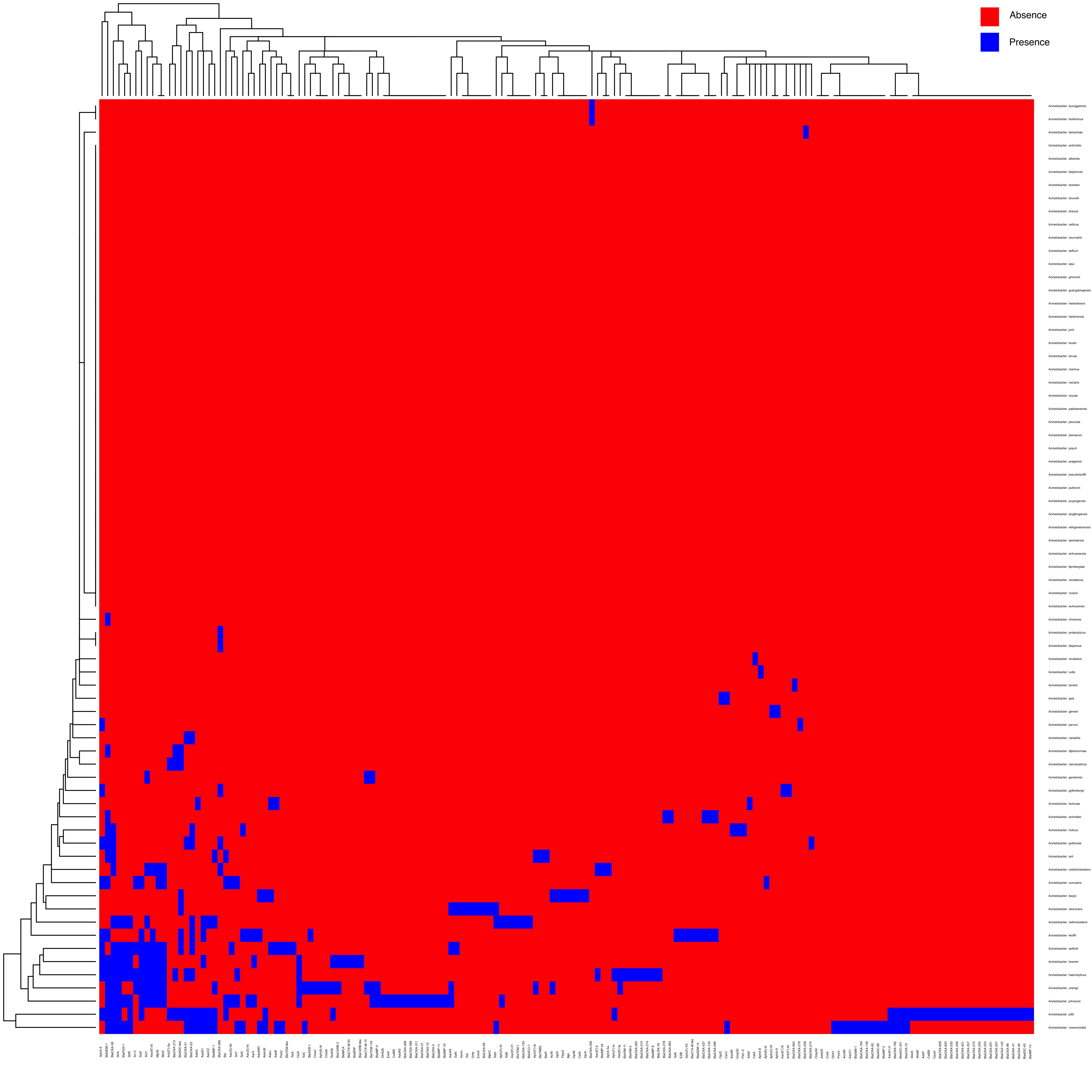
Absence
Presence
Acinetobacter kyonggiensis
Acinetobacter bohemicus
Acinetobacter bereziniae
Acinetobacter antiviralis
Acinetobacter albensis
Acinetobacter beijerinckii
Acinetobacter boissieri
Acinetobacter bouvetii
Acinetobacter brisouii
Acinetobacter celticus
Acinetobacter courvalinii
Acinetobacter defluvii
Acinetobacter equi
Acinetobacter grimontii
Acinetobacter guangdongensis
Acinetobacter halotolerans
Acinetobacter harbinensis
Acinetobacter junii
Acinetobacter kookii
Acinetobacter larvae
Acinetobacter marinus
Acinetobacter nectaris
Acinetobacter oryzae
Acinetobacter pakistanensis
Acinetobacter piscicola
Acinetobacter plantarum
Acinetobacter populi
Acinetobacter pragensis
Acinetobacter pseudolwoffii
Acinetobacter pullorum
Acinetobacter puyangensis
Acinetobacter qingfengensis
Acinetobacter refrigeratorensis
Acinetobacter seohaensis
Acinetobacter sichuanensis
Acinetobacter tjernbergiae
Acinetobacter venetianus
Acinetobacter vivianii
Acinetobacter wuhouensis
Acinetobacter chinensis
Acinetobacter proteolyticus
Acinetobacter dispersus
Acinetobacter modestus
Acinetobacter rudis
Acinetobacter tandoii
Acinetobacter apis
Acinetobacter gerneri
Acinetobacter parvus
Acinetobacter variabilis
Acinetobacter dijkshoorniae
Acinetobacter calcoaceticus
Acinetobacter gandensis
Acinetobacter gyllenbergii
Acinetobacter lactucae
Acinetobacter schindleri
Acinetobacter indicus
Acinetobacter guillouiae
Acinetobacter soli
Acinetobacter colistiniresistens
Acinetobacter cumulans
Acinetobacter baylyi
Acinetobacter oleivorans
Acinetobacter radioresistens
Acinetobacter lwoffii
Acinetobacter seifertii
Acinetobacter towneri
Acinetobacter haemolyticus
Acinetobacter ursingii
Acinetobacter johnsonii
Acinetobacter pittii
Acinetobacter nosocomialis
